# Hyperleptinemia is associated with parameters of low-grade systemic inflammation and metabolic dysfunction in obese human beings

**DOI:** 10.3389/fnint.2013.00062

**Published:** 2013-08-23

**Authors:** Sonia Leon-Cabrera, Lourdes Solís-Lozano, Karina Suárez-Álvarez, Antonio González-Chávez, Yadira L. Béjar, Guillermo Robles-Díaz, Galileo Escobedo

**Affiliations:** ^1^Departamento de Biología de la Reproducción y Clínica de Desórdenes de Sueño, Universidad Autónoma Metropolitana-IztapalapaD.F., México; ^2^Laboratorio de Hígado, Páncreas y Motilidad, Unidad de Medicina Experimental, Hospital General de MéxicoD.F., México; ^3^Facultad de Medicina, Departamento de Medicina Experimental, Hospital General de México, Universidad Nacional Autónoma de MéxicoD.F., México; ^4^Servicio de Medicina Interna, Hospital General de MéxicoD.F., México; ^5^Servicio de Banco de Sangre, Hospital General de MéxicoD.F., México

**Keywords:** leptin, hyperleptinemia, obesity, low-grade inflammation, metabolic disease, type 2 diabetes, human

## Abstract

Leptin is an adipose tissue-derived hormone that has been involved in hypothalamic and systemic inflammation, altered food-intake patterns, and metabolic dysfunction in obese mice. However, it remains unclear whether leptin has a relationship with parameters of systemic inflammation and metabolic dysfunction in humans. We thus evaluated in a cross-sectional study the circulating levels of leptin in 40 non-obese and 41 obese Mexican individuals, examining their relationship with tumor necrosis factor alpha (TNF-α), interleukin (IL) 12, IL-10, central obesity, serum glucose and insulin levels, and serum triglyceride and cholesterol concentrations. Circulating levels of leptin, TNF-α, IL-12, IL-10, and insulin were measured by ELISA, while concentrations of glucose, triglyceride, and cholesterol were determined by enzymatic assays. As expected, serum levels of leptin exhibited a significant elevation in obese individuals as compared to non-obese subjects, showing a clear association with increased body mass index (*r* = 0.4173), central obesity (*r* = 0.4678), and body fat percentage (*r* = 0.3583). Furthermore, leptin also showed a strong relationship with serum TNF-α (*r* = 0.6989), IL-12 (*r* = 0.3093), and IL-10 (*r* = −0.5691). Interestingly, leptin was also significantly related with high concentrations of fasting glucose (*r* = 0.5227) and insulin (*r* = 0.2229), as well as elevated levels of insulin resistance (*r* = 0.3611) and circulating triglyceride (*r* = 0.4135). These results suggest that hyperleptinemia is strongly associated with the occurrence of low-grade systemic inflammation and metabolic alteration in obese subjects. Further clinical research is still needed to determine whether hyperleptinemia may be a potential marker for recognizing the advent of obesity-related metabolic disorders in human beings.

## Introduction

Obesity is now considered a major health problem worldwide, with a growing prevalence in the Mexican population (Olaiz-Fernández et al., 2006; Popkin, 2011). Obese people show a higher risk to develop type 2 diabetes (T2D), coronary heart disease, stroke, arterial hypertension, non-alcoholic steatohepatitis (NASH), and other obesity-related metabolic disorders (Ritchie and Connell, 2007). These pathologies have been recently associated with a low-grade systemic inflammatory state (Odegaard and Chawla, 2008), characterized by altered circulating levels of inflammatory mediators in the obese subject, including tumor necrosis factor alpha (TNF-α), interleukin (IL-) 12, C-reactive protein (CRP), and IL-10 (Rush et al., 2007; Bremer et al., 2011). TNF-α has been shown to increase with adiposity in mice and human beings (Steinberg et al., 2006). Circulating concentrations of IL-12 and CRP are elevated in overweight and obese individuals, exhibiting a significant association with increased body mass index (BMI) and waist circumference, as well high glucose and triglyceride levels (Visser et al., 1999; Suarez-Alvarez et al., 2013). On the contrary, serum IL-10 levels have been shown to decrease in high-fat diet fed-mice (Gotoh et al., 2012). As it can be seen, circulating proinflammatory factors have received growing attention since they could play a major role in mediating the low-grade inflammatory state, which seems to decisively contribute to the advent of obesity-related metabolic disorders (Ritchie and Connell, 2007; Bertola et al., 2010).

Leptin is an adipose tissue-derived hormone belonging to the class-I helical cytokine family (Trinchieri, 2003). Leptin has been shown to regulate food-intake and energy expenditure in both rodents and humans (Houseknecht et al., 1998). Interestingly, circulating levels of leptin have been shown to increase in high-fat diet fed-mice and obese subjects, leading to a state of hyperleptinemia (Maffei et al., 1995; Lin et al., 2000). However, although hyperleptinemia strongly correlates with parameters of low-grade systemic inflammation and metabolic dysfunction in animal models of obesity (Munzberg, 2008; Arruda et al., 2011; Stienstra et al., 2011), they show controversial results in human beings. For instance, it has been reported that serum leptin is augmented in obese individuals with metabolic syndrome (MetS) that also show an elevation in the plasma levels of CRP (Kim et al., 2006). In contrast, obese non-diabetic women subjected to a caloric restriction diet show decreased values in plasma leptin without exhibiting a significant diminution in the circulating levels of TNF-α (Agueda et al., 2012). In the same sense, in obese adolescents leptin has been shown to rise independently of the levels of insulin resistance and TNF-α (Aguilar et al., 2012; Cohen et al., 2012). As it can be seen, it is still unclear whether hyperleptinemia has an association with the occurrence of low-grade systemic inflammation and metabolic dysfunction in humans.

We thus studied the serum levels of leptin in non-obese and obese Mexican individuals, examining their possible relationship with parameters of low-grade systemic inflammation (TNF-α, IL-12, and IL-10) and metabolic alteration (elevated serum glucose and insulin, increased level of insulin resistance, high triglyceride and cholesterol concentrations, as well as increasing waist circumference and body fat percentage).

## Materials and methods

### Subjects

A total of 81 apparently healthy Mexican adult volunteers from the south-central region of Mexico were included in the study. All of the participants provided written informed consent, previously approved by an institutional review board of the General Hospital of Mexico “Dr. Eduardo Liceaga,” which guaranteed that the study was conducted in accordance with the principles described at the Helsinki Declaration. Subjects were excluded from the study if they had previous or recent diagnosis of diabetes mellitus, cardiovascular diseases, chronic hepatic or renal disease, blood pressure higher than 140/90 mm Hg, inflammatory or autoimmune disorders, acute or chronic infectious diseases, cancer, and endocrine disorders. We additionally excluded pregnant or lactating women, subjects under any kind of cardiometabolic medication including anti-inflammatory, anti-aggregant, and anti-hypertensive drugs, and subjects without having an 8–12 h overnight fasting. All of the individuals enrolled into the study received full medical evaluation, including the achievement of clinic history and physical examination by a physician.

### Measurement of anthropometric parameters

According to the World Health Organization criteria for BMI, all of the participants were divided into two groups: control non-obese subjects (BMI 18.5–24.9 kg/m^2^) and obese subjects (BMI ≥ 30 kg/m^2^), where BMI resulted of dividing weight by height squared (kg/m^2^). Waist circumference was obtained from each study subject, considering the midpoint between the lower rib margin and the iliac crest, using a conventional tape in centimeters (cm). For women, abdominal obesity was considered when their waist circumference were 80 cm or higher, whereas for men it was considered when their waist circumference were 94 cm or higher. Percentage of body fat was individually recorded by using a body composition analyzer (TANITA®, Body Composition Analyzer, Model TBF-300A, Tokyo, Japan).

### Measurement of metabolic parameters

Blood samples were individually taken after overnight fasting, and collected into pyrogen-free tubes (VacutainerTM, BD Diagnostics, NJ, USA) at room temperature. Collection tubes were then centrifuged at 1000 g/4°C for 30 min, and serum samples obtained and stored at −80°C in numerous aliquots until use. Total cholesterol and triglyceride were individually measured in triplicate by an enzymatic assay according to manufacturer's instructions (Roche Diagnostics, Mannhein, Germany). Serum insulin levels were individually determined in triplicate by means of the enzyme-linked immunosorbent assay (ELISA), following the manufacturer's instructions (Abnova Corporation, Taiwan). Serum glucose levels were individually determined in triplicate by the glucose oxidase assay, following the manufacturer's instructions (Megazyme International, Ireland). The estimate of insulin resistance was individually determined by means of the HOMA-IR, as follows: fasting insulin concentration (mU/L) × fasting glucose concentration (mmol/L) divided by 22.5.

### Measurement of leptin and low-grade systemic inflammation parameters

Blood samples were individually taken after overnight fasting, and collected into pyrogen-free tubes (VacutainerTM, BD Diagnostics, NJ, USA) at room temperature. Collection tubes were then centrifuged at 1000 g/4°C for 30 min, and serum samples obtained and stored at −80°C in numerous aliquots until use. Serum levels of leptin, TNF-α, IL-10, and IL-12 were determined in triplicate by ELISA, following the manufacturer's instructions (Peprotech, Mexico).

### Statistical analysis

Data from anthropometric and metabolic parameters were analyzed by using the Student's *t*-test for determining significant differences. Data from leptin, TNF-α, IL-10, and IL-12 were analyzed by means of using the Mann-Whitney *U*-test for determining significant differences. The Spearman's correlation coefficient was performed for examining the relationship of leptin with anthropometric, metabolic, and inflammatory parameters. All of the studied groups were matched by gender and age. Statistical analysis was performed using the GraphPad Prism 5 software. Differences were considered significant when *p* < 0.05.

## Results

A total of 40 non-obese controls and 41 obese subjects were included in the study. No significant differences were observed in age (for non-obese controls mean age 29.9 ± 10.35 years, whereas for obese subjects mean age 34.9 ± 10.24 years), and women/men proportion (22 women and 18 men in the non-obese control group, and 20 women and 21 men in the obesity group) in the studied groups (Table 1). In contrast, BMI, waist circumference, body fat percentage, fasting blood glucose and insulin, circulating levels of triglyceride and cholesterol, and serum concentrations of TNF-α and IL-12 were clearly increased in obese individuals as comparing with non-obese control subjects (Table 1). Concomitantly, serum levels of IL-10 were significantly lower in obese individuals than in non-obese subjects (Table 1). It merits to mention that BMI was clearly correlated with waist circumference (*r* = 0.9297, *p* < 0.0001) and body fat percentage (*r* = 0.7655, *p* < 0.0001) in our study population. In a similar way, there was also a significant association between waist circumference and body fat percentage (*r* = 0.7655, *p* < 0.0001).

**Table 1:** **Anthropometric, metabolic, and inflammatory characteristics of the study subjects**.

	**Non-obese**	**Obese**	***P*-value**
Gender (W/M)	22/18	20/21	N.S.
Age (years)	29.9 ± 10.3	34.9 ± 10.2	N.S.
BMI (kg/m^2^)	22.6 ± 1.8	33.7 ± 3.4	*p* < 0.0001
Waist circumference (cm)	79.6 ± 6.5	107.4 ± 9.9	*p* < 0.0001
Body fat percentage	24.6 ± 8.2	37.6 ± 6.9	*p* < 0.05
Fasting blood glucose (mmol/L)	4.3 ± 0.1	5.89 ± 0.3	*p* < 0.0001
Fasting blood insulin (mU/L)	12.6 ± 1.4	15.5 ± 6.7	*p* = 0.0088
HOMA-IR	2.4 ± 0.3	4.08 ± 1.8	*p* < 0.0001
Total cholesterol (mg/dL)	192.8 ± 10.1	198.1 ± 10.3	*p* = 0.0219
Total triglyceride (mg/dL)	137.5 ± 9.1	251.9 ± 14.4	*p* < 0.0001
TNF-α (pg/mL)	271.8 ± 28.05	322.9 ± 58.5	*p* < 0.05
IL-12 (pg/mL)	272.9 ± 13.6	381.5 ± 59.8	*p* < 0.0001
IL-10 (pg/mL)	1145.2 ± 214.6	840.8 ± 96.5	*p* < 0.0001

*Abbreviations: W, women; M, men; BMI, body mass index; HOMA-IR, homeostatic model assessment-insulin resistance; TNF-α, tumor necrosis factor alpha; IL, interleukin; N.S., non-significant differences*.

*Data are presented as mean ± standard deviation*.

*Differences were considered significant when p < 0.05*.

**Figure 1:**
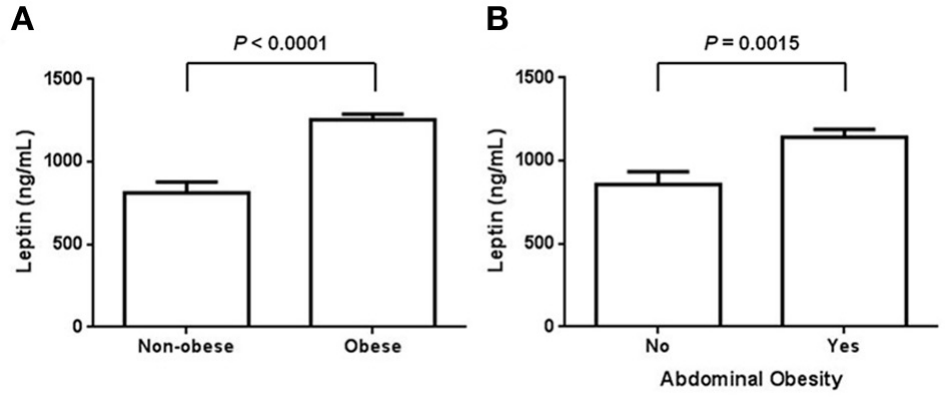
**Serum levels of leptin in obese and non-obese individuals. (A)** Circulating leptin levels were assessed in normal weight and obese subjects, defining obesity according to the World Health Organization criteria for body mass index. In our study population, serum leptin was also evaluated in terms of abdominal obesity **(B)**. For women, abdominal obesity was considered when the waist circumference was 80 cm or higher, whereas for men, it was considered when the waist circumference was 94 cm or higher. Data are expressed as mean ± S. E. Differences were considered significant when *p* < 0.05.

In accordance with previous reports, circulating levels of leptin were significantly increased in obese subjects when comparing to non-obese control individuals. In terms of BMI, leptin exhibited a significant 1.5-fold increase in obese subjects as comparing with normal weight controls (Figure 1A). In obese individuals, the mean value of leptin was 1256.1 ± 207.7 ng/mL, whereas in the non-obese group it was around 812.1 ± 417.8 ng/mL (Figure 1A). Interestingly, serum values of leptin still showed a significant elevation when examining in subjects with abdominal obesity (Figure 1B). For this case, the mean value of leptin in subjects with abdominal obesity was 1141.8 ± 343.6 ng/mL, while it decreased to 858.3 ± 419.6 ng/mL in individuals exhibiting a normal waist perimeter (Figure 1B).

**Figure 2:**
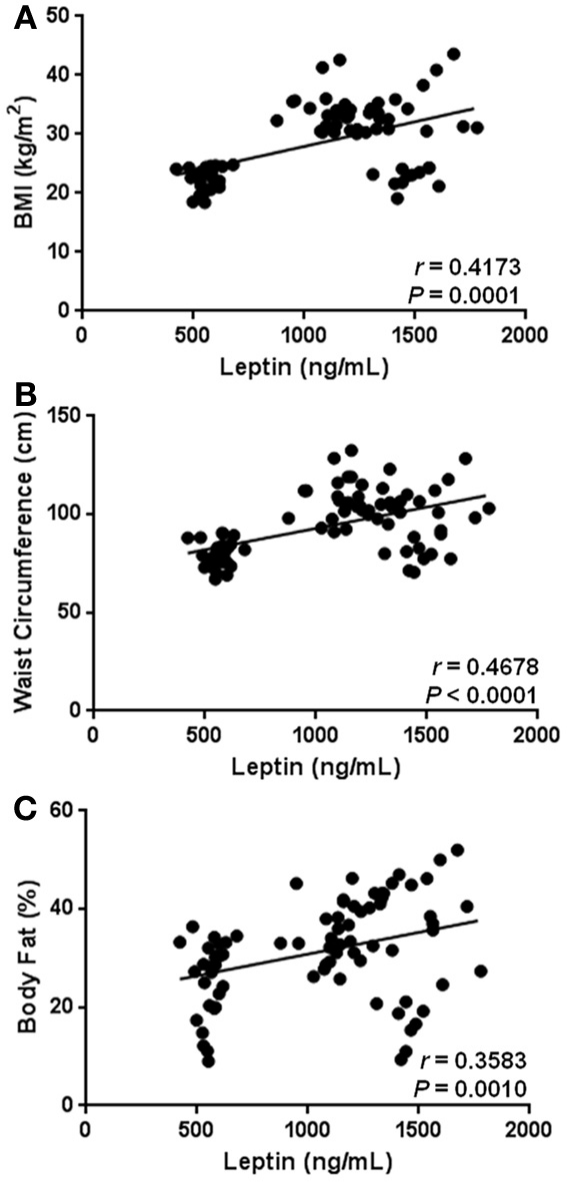
**Statistical correlation between serum levels of leptin and anthropometric parameters of obesity.** Serum levels of leptin were positively associated with body mass index (BMI) **(A)**, waist circumference **(B)**, and body fat percentage **(C)**. Coefficients (*r*) and *P*-values were calculated by using the Spearman's correlation model. The correlation level was considered significant when *p* < 0.05.

**Figure 3:**
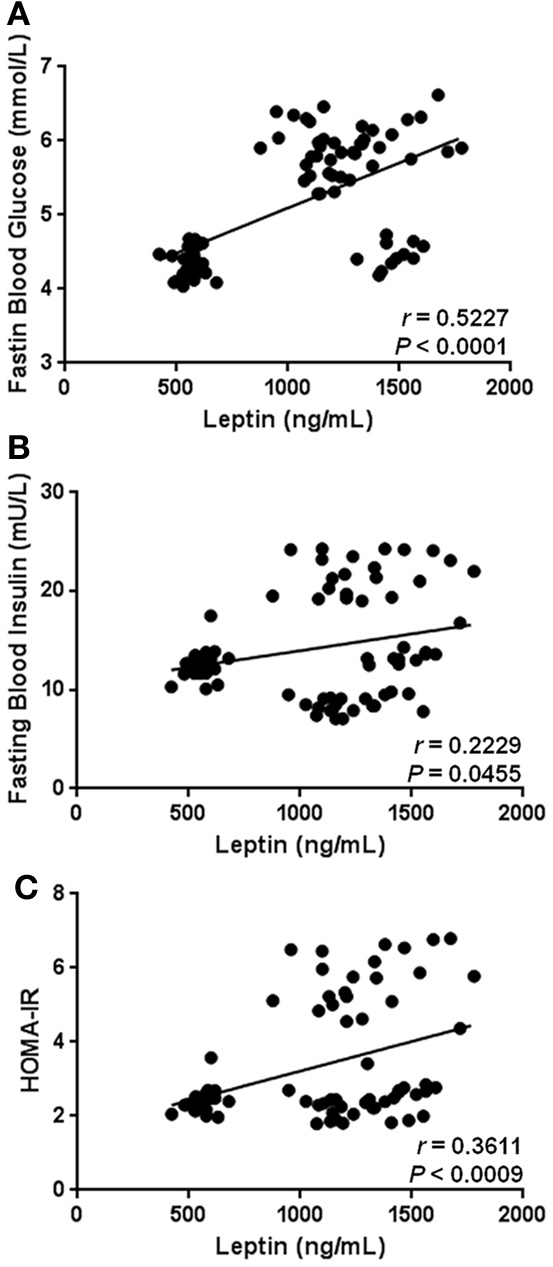
**Statistical correlation of the serum levels of leptin with fasting blood glucose, fasting blood insulin, and insulin resistance.** Serum concentrations of leptin exhibited a positive significant relationship with high levels of glucose **(A)** and insulin **(B)**, as well increased insulin resistance **(C)**. The level of insulin resistance was estimated using the HOMA-IR index, which results of multiplying fasting insulin concentration (mU/L) by fasting glucose concentration (mmol/L), then divided by the constant 22.5. Coefficients (*r*) and *P*-values were calculated by the Spearman's correlation model. The correlation level was considered significant when *p* < 0.05.

**Figure 4:**
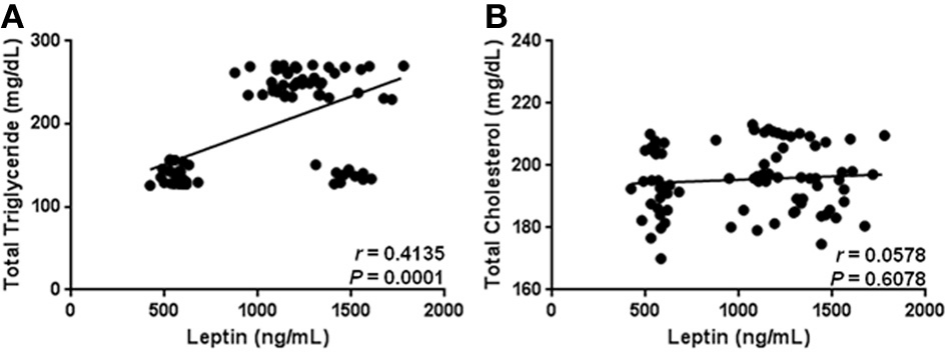
**Statistical correlation of the serum levels of leptin with total triglyceride and cholesterol levels.** Serum concentrations of leptin showed a positive significant relationship with high levels of triglycerides **(A)**, but not with total cholesterol **(B)**. Coefficients (*r*) and *P*-values were calculated by the Spearman's correlation model. The correlation level was considered significant when *p* < 0.05.

As expected, our results show that circulating levels of leptin increase with obesity-related anthropometric parameters. Indeed, leptin was significantly correlated with increased BMI (*r* = 0.4173, *p* = 0.0001), central obesity (*r* = 0.4678, *p* < 0.0001), and body fat percentage (*r* = 0.3583, *p* = 0.0010) (Figures 2A–C, respectively). Furthermore, leptin also exhibited a clear association with parameters of metabolic alteration. In this sense, serum leptin was significantly related with increased levels of blood glucose (*r* = 0.5227, *p* < 0.0001) and insulin (*r* = 0.2229, *p* = 0.0455) (Figures 3A,B, respectively). There was also a significant relationship between leptin and the level of insulin resistance, estimated by means of the HOMA-IR (*r* = 0.3611, *p* < 0.0009) (Figure 3C). Interestingly, leptin had a positive association with increased triglyceride levels (*r* = 0.4135, *p* = 0.0001), but not with total cholesterol (Figures 4A,B, respectively).

**Figure 5:**
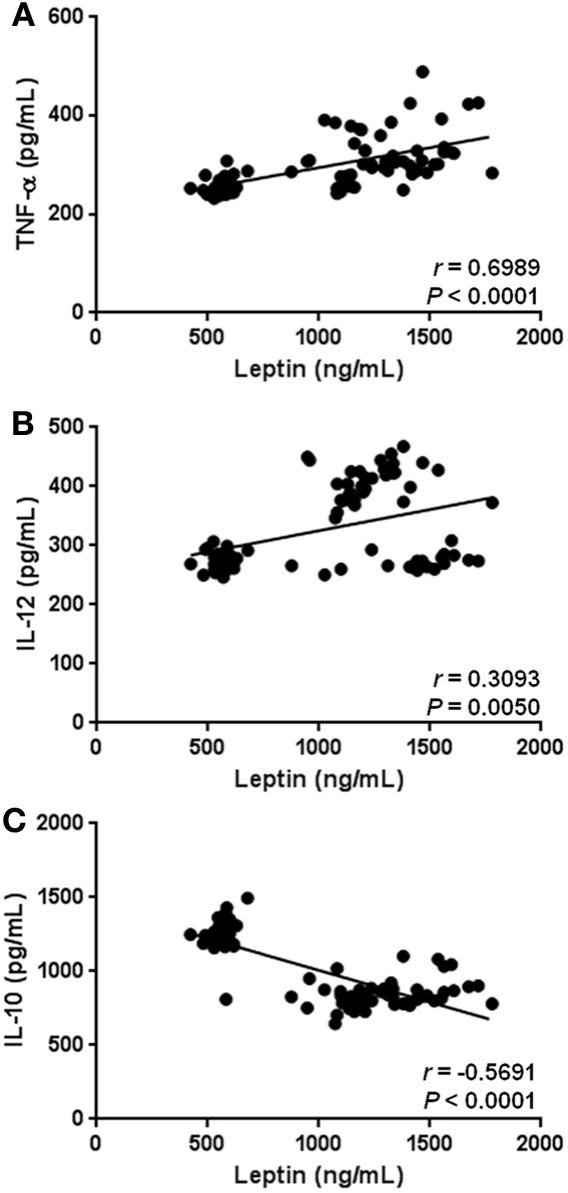
**Statistical correlation of the serum levels of leptin with parameters of low-grade systemic inflammation.** Serum concentrations of leptin showed a positive significant relationship with increased serum levels of TNF-α **(A)**, and IL-12 **(B)**. At the same time, hyperleptinemia exhibited a significant inverse association with circulating concentrations of IL-10 **(C)**. Coefficients (*r*) and *P*-values were calculated by the Spearman's correlation model. The correlation level was considered significant when *p* < 0.05.

Besides having significant relationships with anthropometric and biochemical parameters associated with obesity-related metabolic alterations, hyperleptinemia also showed a strong relation with markers of low-grade systemic inflammation in the study subjects. In fact, circulating leptin was positively correlated with serum levels of TNF-α (*r* = 0.6989, *p* < 0.0001) and IL-12 (*r* = 0.3093, *p* = 0.0050) (Figures 5A,B), whereas a significant negative relationship between leptin and IL-10 was observed in the study population (*r* = −0.5691, *p* < 0.0001) (Figure 5C).

## Discussion

As mentioned, it has been consistently shown that serum concentrations of leptin increase in high-fat diet fed-mice and obese humans. In mice, hyperleptinemia is related to hyperphagia and fat depot augmentation, while it is associated with increased white adipose tissue (WAT) mass and body weight gain in human beings (Stanley et al., 2005). However, recent experimental evidence from animal models of obesity suggests that leptin is not only a marker of weight gain but also seems to have a relationship with developing a systemic state of low-grade inflammation and metabolic disturbance. In fact, obese mice exhibiting hyperleptinemia show increased plasma levels of inflammatory cytokines (Dube et al., 2008; Yang et al., 2010; Enos et al., 2013), accompanied by numerous metabolic disorders including hyperglycemia (Yang et al., 2010), hyperinsulinemia (Stienstra et al., 2011), hyperlipidemia (Kang et al., 2011), liver steatosis (Shih et al., 2010), and insulin resistance (Yang et al., 2010). Taking this experimental evidence into account, it is important to evaluate whether hyperleptinemia could also be associated to the establishment of systemic inflammation and metabolic dysfunction in human beings with high metabolic risk, such as obese individuals.

In humans, the relationship of leptin with a state of metabolic dysfunction has been barely studied, showing inconclusive results to date. Indeed, a study conducted in a group of obese adolescents revealed that high serum levels of leptin correlate with increased BMI and waist circumference, without having a significant relation with the insulin resistance level (Aguilar et al., 2012). In contrast, recent data from a cross-sectional survey conducted in obese adults demonstrated that hyperleptinemia is clearly associated with BMI, hyperinsulinemia, and insulin resistance (Martins Mdo et al., 2012). Our results are consistent with the last study, since high levels of leptin were significantly correlated with hyperglycemia, hyperinsulinemia, increased HOMA-IR, and hypertriglyceridemia in our study population. A possible explanation to understand this apparently controversial finding may involve the age of the subjects included in each study. As it is widely known, the level of sex-steroid hormones reaches a plateau during maturity, in comparison to both childhood and adolescence where numerous hormonal variations are normally observed (Stanhope and Brook, 1988; Rogol, 2004). Interestingly, it has been recently reported that sex-steroid hormones are able to upregulate the leptin expression in human and rat cells (Feng et al., 2011; Gambino et al., 2012). Therefore, it is feasible to expect that synthesis of leptin may be enhanced during adulthood, which may contribute to decrease the leptin levels in children/adolescents in comparison with adults. However, additional clinical studies considering the influence of sex-steroid hormones upon the systemic levels of leptin are necessary in order to address major conclusions.

An interesting finding in this cross-sectional study involves the relationship of hyperleptinemia with a systemic state of low-grade inflammation in obese human beings. A recent study demonstrated that leptin is overexpressed in the subcutaneous adipose tissue (SAT) of obese individuals with MetS, as comparing with SAT from healthy obese subjects and non-obese individuals (Farb et al., 2011). Furthermore, increasing in the leptin expression is accompanied by macrophage infiltration and overexpression of proinflammatory cytokines such as IL-1β, IL-6, and IL-8 in the SAT of those same patients (Bremer et al., 2011; Farb et al., 2011). Consistent with this previous study, our results show that hyperleptinemia is significantly associated with high serum levels of TNF-α and IL-12, as well as reduced concentrations of IL-10 in subjects with central obesity, hyperglycemia, increased insulin resistance, and hypertriglyceridemia. IL-12 is a cytokine with the ability to induce synthesis of interferon-gamma (IFN-γ) in T cells and natural killer cells (Trinchieri, 2003). IFN-γ is a key mediator in releasing of TNF-α by classically activated macrophages (Odegaard and Chawla, 2008). Taking into consideration that serum IFN-γ has been shown to increase during obesity (Azar Sharabiani et al., 2011), it is conceivable to expect a positive relationship among TNF-α, IL-12, and leptin in our study population. Another intriguing issue concerning the positive association among leptin, TNF-α, and IL-12, involves the ability of leptin to regulate the expression of inflammatory cytokines. It has been recently reported that leptin is able to stimulate the *in vitro* production of TNF-α and IL-1β in human mononuclear cells (Tsiotra et al., 2013). Thus, it is now proposed that high levels of leptin may induce the production of proinflammatory cytokines in obese people, contributing in this way to the systemic inflammation observed in these subjects. Nevertheless, before establishing a possible cause-and-effect relation among leptin, TNF-α, and IL-12 in the scenario of obesity, additional prospective clinical research is required. Moreover, consistent with the installation of a systemic state of low-grade inflammation, we observed a significant reduction in the circulating levels of IL-10 in obese individuals as comparing with non-obese subjects. IL-10 is a cytokine with potent anti-inflammatory properties in mice and humans (Saraiva and O'garra, 2010). However, the role of IL-10 during systemic inflammation and metabolic dysfunction is still poorly understood (Formoso et al., 2012; Tajik et al., 2012). For this reason, it is important to mention that the present work is one of the first contributions showing a significant inverse correlation between serum IL-10 and hyperleptinemia in obese individuals. Collectively, these findings suggest that obesity-related hyperleptinemia is accompanied by a low-grade inflammatory profile, characterized by increased circulating levels of TNF-α and IL-12, and reduced concentrations of IL-10. Whether hyperleptinemia is cause or consequence of the systemic inflammatory milieu in humans, is a matter worthy of being considered in further basic and clinical studies.

Present results demonstrate that high circulating levels of leptin are significantly associated with a systemic state of low-grade inflammation and metabolic dysfunction in obese subjects. Additional prospective clinical studies are still required to evaluate whether hyperleptinemia may be used as a marker for recognizing the advent of obesity-related metabolic disorders in human beings.

### Conflict of interest statement

The authors declare that the research was conducted in the absence of any commercial or financial relationships that could be construed as a potential conflict of interest.

## References

[B1] Agueda M. Lasa A. Simon E. Ares R. Larrarte E. Labayen I. (2012). Association of circulating visfatin concentrations with insulin resistance and low-grade inflammation after dietary energy restriction in Spanish obese non-diabetic women: role of body composition changes. Nutr. Metab. Cardiovasc. Dis. 22, 208–214 10.1016/j.numecd.2010.06.010 20951014

[B2] Aguilar M. J. Gonzalez-Jimenez E. Antelo A. Perona J. S. (2012). Insulin resistance and inflammation markers: correlations in obese adolescents. J. Clin. Nurs. 22, 2002–2010 10.1111/jocn.12034 23216620

[B3] Arruda A. P. Milanski M. Coope A. Torsoni A. S. Ropelle E. Carvalho D. P. (2011). Low-grade hypothalamic inflammation leads to defective thermogenesis, insulin resistance, and impaired insulin secretion. Endocrinology 152, 1314–1326 10.1210/en.2010-0659 21266511

[B4] Azar Sharabiani M. T. Vermeulen R. Scoccianti C. Hosnijeh F. S. Minelli L. Sacerdote C. (2011). Immunologic profile of excessive body weight. Biomarkers 16, 243–251 10.3109/1354750X.2010.547948 21506696

[B5] Bertola A. Bonnafous S. Anty R. Patouraux S. Saint-Paul M. C. Iannelli A. (2010). Hepatic expression patterns of inflammatory and immune response genes associated with obesity and NASH in morbidly obese patients. PLoS ONE 5:e13577 10.1371/journal.pone.0013577 21042596 PMC2962651

[B6] Bremer A. A. Devaraj S. Afify A. Jialal I. (2011). Adipose tissue dysregulation in patients with metabolic syndrome. J. Clin. Endocrinol. Metab. 96, E1782–E1788 10.1210/jc.2011-1577 21865369 PMC3205887

[B7] Cohen J. I. Maayan L. Convit A. (2012). Preliminary evidence for obesity-associated insulin resistance in adolescents without elevations of inflammatory cytokines. Diabetol. Metab. Syndr. 4, 26 10.1186/1758-5996-4-26 22682228 PMC3509401

[B8] Dube M. G. Torto R. Kalra S. P. (2008). Increased leptin expression selectively in the hypothalamus suppresses inflammatory markers CRP and IL-6 in leptin-deficient diabetic obese mice. Peptides 29, 593–598 10.1016/j.peptides.2008.01.001 18325632 PMC2291149

[B9] Enos R. T. Davis J. M. Velazquez K. T. Mcclellan J. L. Day S. D. Carnevale K. A. (2013). Influence of dietary saturated fat content on adiposity, macrophage behavior, inflammation, and metabolism: composition matters. J. Lipid Res. 54, 152–163 10.1194/jlr.M030700 23103474 PMC3520521

[B10] Farb M. G. Bigornia S. Mott M. Tanriverdi K. Morin K. M. Freedman J. E. (2011). Reduced adipose tissue inflammation represents an intermediate cardiometabolic phenotype in obesity. J. Am. Coll. Cardiol. 58, 232–237 10.1016/j.jacc.2011.01.051 21737012 PMC3132399

[B11] Feng Y. Shao R. Weijdegard B. Wang T. Johansson J. Sun S. (2011). Effects of androgen and leptin on behavioral and cellular responses in female rats. Horm. Behav. 60, 427–438 10.1016/j.yhbeh.2011.07.012 21819988

[B12] Formoso G. Taraborrelli M. Guagnano M. T. D'adamo M. Di Pietro N. Tartaro A. (2012). Magnetic resonance imaging determined visceral fat reduction associates with enhanced IL-10 plasma levels in calorie restricted obese subjects. PLoS ONE 7:e52774 10.1371/journal.pone.0052774 23300769 PMC3530499

[B13] Gambino Y. P. Perez Perez A. Duenas J. L. Calvo J. C. Sanchez-Margalet V. Varone C. L. (2012). Regulation of leptin expression by 17beta-estradiol in human placental cells involves membrane associated estrogen receptor alpha. Biochim. Biophys. Acta 1823, 900–910 10.1016/j.bbamcr.2012.01.015 22310000

[B14] Gotoh K. Inoue M. Masaki T. Chiba S. Shimasaki T. Ando H. (2012). A novel anti-inflammatory role for spleen-derived interleukin-10 in obesity-induced hypothalamic inflammation. J. Neurochem. 120, 752–764 10.1111/j.1471-4159.2011.07617.x 22146087

[B15] Houseknecht K. L. Baile C. A. Matteri R. L. Spurlock M. E. (1998). The biology of leptin: a review. J. Anim. Sci. 76, 1405–1420 9621947 10.2527/1998.7651405x

[B16] Kang J. S. Lee W. K. Yoon W. K. Kim N. Park S. K. Park H. K. (2011). A combination of grape extract, green tea extract and L-carnitine improves high-fat diet-induced obesity, hyperlipidemia and non-alcoholic fatty liver disease in mice. Phytother. Res. 25, 1789–1795 10.1002/ptr.3476 21480410

[B17] Kim C. S. Park H. S. Kawada T. Kim J. H. Lim D. Hubbard N. E. (2006). Circulating levels of MCP-1 and IL-8 are elevated in human obese subjects and associated with obesity-related parameters. Int. J. Obes. 30, 1347–1355 10.1038/sj.ijo.0803259 16534530

[B18] Lin S. Thomas T. C. Storlien L. H. Huang X. F. (2000). Development of high fat diet-induced obesity and leptin resistance in C57Bl/6J mice. Int. J. Obes. Relat. Metab. Disord. 24, 639–646 10.1038/sj.ijo.0801209 10849588

[B19] Maffei M. Halaas J. Ravussin E. Pratley R. E. Lee G. H. Zhang Y. (1995). Leptin levels in human and rodent: measurement of plasma leptin and ob RNA in obese and weight-reduced subjects. Nat. Med. 1, 1155–1161 10.1038/nm1195-1155 7584987

[B20] Martins Mdo C. Lima Faleiro L. Fonseca A. (2012). [Relationship between leptin and body mass and metabolic syndrome in an adult population]. Rev. Port. Cardiol. 31, 711–719 23040870 10.1016/j.repc.2012.08.002

[B21] Munzberg H. (2008). Differential leptin access into the brain—a hierarchical organization of hypothalamic leptin target sites? Physiol. Behav. 94, 664–669 10.1016/j.physbeh.2008.04.020 18502454

[B22] Odegaard J. I. Chawla A. (2008). Mechanisms of macrophage activation in obesity-induced insulin resistance. Nat. Clin. Pract. Endocrinol. Metab. 4, 619–626 10.1038/ncpendmet0976 18838972 PMC3381907

[B23] Olaiz-Fernández G. Rivera-Dommarco J. Shamah-Levy T. Rojas R. Villalpando-Hernández S. Hernández-Avila M. (2006). Encuesta Nacional de Salud y Nutrición 2006. Cuernavaca: Instituto Nacional de Salud Pública

[B24] Popkin B. M. (2011). Does global obesity represent a global public health challenge? Am. J. Clin. Nutr. 93, 232–233 10.3945/ajcn.110.008458 21159790 PMC3021421

[B25] Ritchie S. A. Connell J. M. (2007). The link between abdominal obesity, metabolic syndrome and cardiovascular disease. Nutr. Metab. Cardiovasc. Dis. 17, 319–326 10.1016/j.numecd.2006.07.005 17110092

[B26] Rogol A. D. (2004). Gender and hormonal regulation of growth. J. Pediatr. Endocrinol. Metab. 17 Suppl 4, 1259–1265 15506071

[B27] Rush E. C. Plank L. D. Yajnik C. S. (2007). Interleukin-6, tumour necrosis factor-alpha and insulin relationships to body composition, metabolism and resting energy expenditure in a migrant Asian Indian population. Clin. Endocrinol. (Oxf) 66, 684–690 10.1111/j.1365-2265.2007.02801.x 17381487

[B28] Saraiva M. O'garra A. (2010). The regulation of IL-10 production by immune cells. Nat. Rev. Immunol. 10, 170–181 10.1038/nri2711 20154735

[B29] Shih C. C. Lin C. H. Wu J. B. (2010). Eriobotrya japonica improves hyperlipidemia and reverses insulin resistance in high-fat-fed mice. Phytother. Res. 24, 1769–1780 10.1002/ptr.3143 20564460

[B30] Stanhope R. Brook C. G. (1988). An evaluation of hormonal changes at puberty in man. J. Endocrinol. 116, 301–305 10.1677/joe.0.1160301 3280720

[B31] Stanley S. Wynne K. Mcgowan B. Bloom S. (2005). Hormonal regulation of food intake. Physiol. Rev. 85, 1131–1158 10.1152/physrev.00015.2004 16183909

[B32] Steinberg G. R. Michell B. J. Van Denderen B. J. Watt M. J. Carey A. L. Fam B. C. (2006). Tumor necrosis factor alpha-induced skeletal muscle insulin resistance involves suppression of AMP-kinase signaling. Cell Metab. 4, 465–474 10.1016/j.cmet.2006.11.005 17141630

[B33] Stienstra R. Van Diepen J. A. Tack C. J. Zaki M. H. Van De Veerdonk F. L. Perera D. (2011). Inflammasome is a central player in the induction of obesity and insulin resistance. Proc. Natl. Acad. Sci. U.S.A. 108, 15324–15329 10.1073/pnas.1100255108 21876127 PMC3174591

[B34] Suarez-Alvarez K. Solis-Lozano L. Leon-Cabrera S. Gonzalez-Chavez A. Gomez-Hernandez G. Quinones-Alvarez M. S. (2013). Serum IL-12 Is increased in Mexican obese subjects and associated with low-grade inflammation and obesity-related parameters. Mediators Inflamm. 2013:967067 10.1155/2013/967067 23533314 PMC3590791

[B35] Tajik N. Keshavarz S. A. Masoudkabir F. Djalali M. Sadrzadeh-Yeganeh H. H. Eshraghian M. R. (2012). Effect of diet-induced weight loss on inflammatory cytokines in obese women. J. Endocrinol. Invest. 36, 211–215 10.3275/8465 22733212

[B36] Trinchieri G. (2003). Interleukin-12 and the regulation of innate resistance and adaptive immunity. Nat. Rev. Immunol. 3, 133–146 10.1038/nri1001 12563297

[B37] Tsiotra P. C. Boutati E. Dimitriadis G. Raptis S. A. (2013). High insulin and leptin increase resistin and inflammatory cytokine production from human mononuclear cells. Biomed Res. Int. 2013:487081 10.1155/2013/487081 23484124 PMC3591160

[B38] Visser M. Bouter L. M. Mcquillan G. M. Wener M. H. Harris T. B. (1999). Elevated C-reactive protein levels in overweight and obese adults. JAMA 282, 2131–2135 10.1001/jama.282.22.2131 10591334

[B39] Yang J. H. Lim H. S. Heo Y. R. (2010). Sasa borealis leaves extract improves insulin resistance by modulating inflammatory cytokine secretion in high fat diet-induced obese C57/BL6J mice. Nutr. Res. Pract. 4, 99–105 10.4162/nrp.2010.4.2.99 20461197 PMC2867230

